# System of Medicine

**Published:** 1880-10

**Authors:** 


A SYSTEM OF MEDICINE. Edited by J. Russell Reynolds, M.D., F.R.S., Fellow of the Royal College of Physicians, London, etc., etc. With numerous additions and illustrations, by Henry Hartshorne, A.M., M.D., Fellow of the College of Physicians, Philadelphia, etc., etc. In three volumes. Vol. III, *Diseases of the Digestive, Blood-Glandular, Urinary, Reproductive and Cutaneous Systems.* Henry C. Lea's Sons & Co., Philadelphia. 1880.

This is the last volume of this very valuable and compendious series. From the necessarily cursory inspection we have given to them, we are led to believe that as a treatise on the Practice of Medicine, the three volumes constituting this series are incomparable. Compendious in detail, and practical and scientific in description, it can but eventually take the first place among present standard works on this subject.

